# Patient derived organoids in prostate cancer: improving therapeutic efficacy in precision medicine

**DOI:** 10.1186/s12943-021-01426-3

**Published:** 2021-09-29

**Authors:** Sahithi Pamarthy, Hatem E. Sabaawy

**Affiliations:** 1 Rutgers Cancer Institute of New Jersey, Rutgers University, 195 Little Albany St, Rm 4557, New Brunswick, NJ 08901 USA; 2 Clinical Investigations and Precision Therapeutics Program, Devision of Medical Oncology, Rutgers, The State University of New Jersey, New Brunswick, NJ 08901 USA; 3 Departments of Pathology and Laboratory Medicine, RBHS-Robert Wood Johnson Medical School, New Brunswick, USA; 4 Departments of Medicine, RBHS-Robert Wood Johnson Medical School, Rutgers, The State University of New Jersey, New Brunswick, NJ 08901 USA

**Keywords:** Prostate cancer, Patient derived organoids, Precision medicine, Targeted therapy

## Abstract

With advances in the discovery of the clinical and molecular landscapes of prostate cancer (PCa), implementation of precision medicine-guided therapeutic testing in the clinic has become a priority. Patient derived organoids (PDOs) are three-dimensional (3D) tissue cultures that promise to enable the validation of preclinical drug testing in precision medicine and coclinical trials by modeling PCa for predicting therapeutic responses with a reliable efficacy. We evaluate the advances in 3D culture and PDO use to model clonal heterogeneity and screen for effective targeted therapies, with a focus on the technological advances in generating PDOs. Recent innovations include the utilization of PDOs both in original research and/or correlative studies in clinical trials to examine drug effects within the PCa tumor microenvironment (TME). There has also been a significant improvement with the utilization of various extracellular matrices and single cell assays for the generation and long-term propagation of PDOs. Single cell derived PDOs could faithfully recapitulate the original tumor and reflect the heterogeneity features. While most PDO use for precision medicine understandably involved tissues derived from metastatic patients, we envision that the generation of PDOs from localized PCa along with the incorporation of cells of the TME in tissue models would fulfill the great potential of PDOs in predicting drug clinical benefits. We conclude that single cell derived PDOs reiterate the molecular features of the original tumor and represent a reliable pre-clinical PCa model to understand individual tumors and design tailored targeted therapies.

## Background

Prostate cancer (PCa) is the second most frequent cancer diagnosed in men worldwide, only behind lung cancer. In 2020, over 1,414,259 new PCa cases and 375,304 deaths were estimated for PCa worldwide [1]. Notably, while the past decade has seen an accelerated decline in the death rate of lung cancer, reduction rate halted for PCa [1].

Androgen receptor signaling plays an active role in the growth and survival of PCa, making medical castration with androgen deprivation therapy (ADT) a mainstay in PCa standard of care. However, many ADT-treated patients develop castration-resistant PCa (CRPC), which is diagnosed by rising levels of prostate-specific antigen despite castration. Current approved treatments for CRPC include hormonal therapy, chemotherapy, immunotherapy, radionuclide therapy, and biomarker based targeted therapies such as PARP inhibitors. Despite several new treatment options, metastatic CRPC (mCRPC) remains a lethal disease with a survival rate below 2–3 years from the time of progression [2].

Effective treatment for PCa in the era of precision oncology relies on predictive molecular signatures to design optimal treatment sequence and combination strategies [3]. Recent studies have identified genomic/transcriptomic signatures capturing the early pathogenetic events in localized PCa (e.g., Ets fusions) and CRPC-associated gene alterations (AR, TP53, PTEN, BRCA 1/2) [4]. However, progress on the use of such molecular-biomarkers for treatment selection in the clinic has been hampered by the limited access to tumor tissue for molecular profiling, the lack of reliable approaches to capture tumor heterogeneity at different disease states, and the imperfect preclinical models [3]. A key for implementing precision medicine in the PCa clinic is to utilize preclinical models that can 1) faithfully recapitulate the cellular, structural, and molecular features of the patient’s tumor, 2) enable drug testing and/or high throughput screening (HTS), and 3) allow the translation of therapy recommendations to the clinic in a timely manner. The use of patient derived organoids (PDOs) could provide an effective strategy to identify optimal drug choices for patients eligible for multiple treatment. In this review, we summarize the past, present, and future of prostate PDOs, outlining how PCa PDOs can be used to improve the therapeutic decision-making process and guide the selection of sequential or combinatorial therapies for the metastatic disease.

### Main text

#### Patient derived preclinical models

Various preclinical models have been used to advance PCa research. Most studies relied on using immortalized cell lines grown in two-dimensional (2D) cultures or engrafted in immunocompromised animals. Despite having these PCa cell lines readily available and simple to use, only a handful exists and with their prolonged culture, they are far from being true representatives of the primary disease. In addition, cell lines fail to capture the various aspects of tumor heterogeneity. Patient-derived xenografts (PDX) are a more superior preclinical models of PCa, since they retain the phenotypic characteristics of the original tumor [5]. While PDXs have the specific advantage of including a microenvironment, they still exist within the limitation of immunocompromised host murine environment and only offer low-throughput therapeutic assessment potential. Furthermore, successful generation of PDXs for drug screening takes several months to accomplish [5, 6]. Moreover, both PDXs and PCa cell lines are frequently derived from the aggressive metastatic disease and therefore there is a paucity of preclinical models for studying primary locoregional PCa.

#### Three-dimensional (3D) culture models in PCa

Various 3D culture models have become popular and considered to be better representative models over 2D cell lines and more amenable to high throughput screening (HTS) assays over PDX models. Overall, 3D cultures allow cancer studies in a close patho-physiological relevance to the tumor growth in the human body. By recreating cancer hallmarks, such as hypoxia, cell-to-cell and/or extracellular matrix (ECM) crosstalk, and complex 3D architectures using biomaterials, these 3D cultures enable the faithful remodeling of the original tumor. Additionally, their suitability for drug HTS has driven their popularity. Morphologically, 3D culture models are multicellular spherical cultures. Based on the starting material and the 3D culture conditions, these models can be largely classified into four types: 1) Spheroid models obtained by culture of immortalized cancer cell lines grown in anchorage independent conditions; 2) Tumorospheres obtained by expansion of cancer stem-like cells (CSCs) in growth factor supplemented serum-free conditions; 3) Organotypic slice cultures, also known as patient derived explants (PDE), obtained by precision-slicing of tumor tissues and grown in gelatin sponges [7], and 4) Organoids, frequently obtained by single cell dissociation or partial dissociation of tumor tissues and grown in serum free medium with biomaterial for ECM support (e.g., matrigel). While the term “prostasphere” has sometimes been used to represent patient derived 3D culture in matrigel, for clarity, we limit the use the term ‘sphere’ or ‘spheroid’ to describe 3D ‘scaffold-free’ cultures, while we reserve the use of the term ‘organoid’ to broadly represent 3D ‘scaffold-based’ multicellular cultures, generated from stem-like and/or organoid forming cells, dissociated from patient derived tissues and cultured in supportive biomaterial for ECM such as matrigel or synthetic hydrogels.

**Fig. 1:**
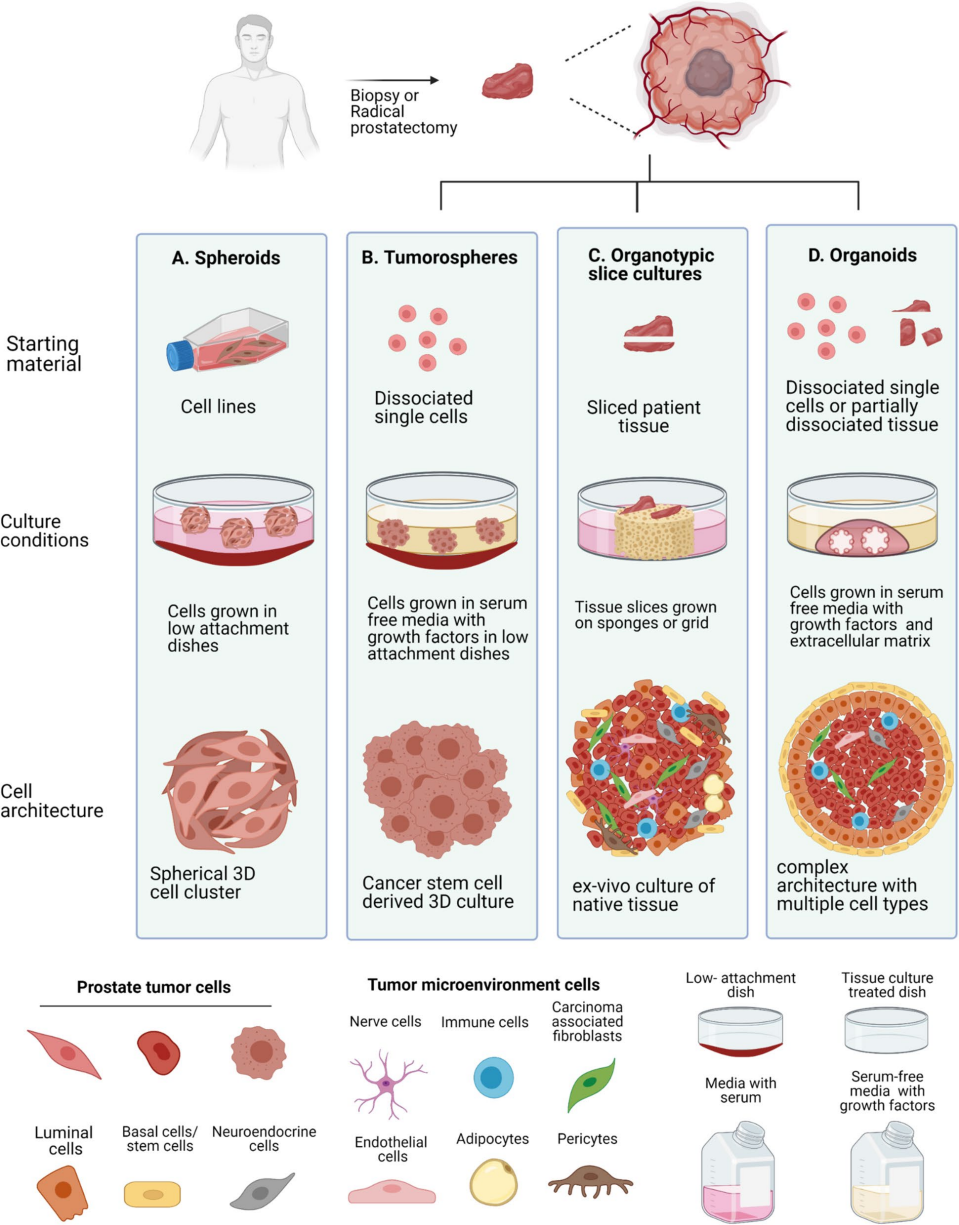
Patient derived in vitro preclinical models of PCa. Based on the starting material and culture conditions, PCa preclinical models are classified into four major types. **A** Spheroids are 3D cultures which constitute established PCa cell lines grown in serum supplemented media within low-attachment dishes/multiwell plates. **B** Tumorospheres are obtained by propagation of dissociated single cancer stem-like cells cultured with growth factors in serum-free media and can be propagated in agar. **C** Organotypic slice cultures are obtained from slicing patient tissue biopsies or surgical specimen and grown on scaffolds such as the wound healing sponge or grids. They are also called patient derived explants (PDEs) and include the cells of tumor microenvironment along with the tumor cells. **D** Organoids are grown in extracellular matrix such as Matrigel embedded droplets supplemented with growth factors and serum-free media. Patient derived organoids (PDOs) represent the tissue architecture of original prostate. The diagram displays different prostate tumor and microenvironmental cell types, culture media, and vessel types. The diagram was created with BioRender.com

Human prostate epithelial cells are enclosed within an ECM and surrounded by various stromal cells including myoepithelial cells, fibroblasts, pericytes, endothelial cells, immune cells, adipocytes, nerve cells, and neuroendocrine cells, constituting the prostate tumor microenvironment (TME) (Fig. 1). While spheroids and tumorospheres do not facilitate PCa cell culture with their surrounding original TME cells, PDEs are short-term ex vivo slice tumor tissue cultures consisting of tumor cells, immune cells, fibroblasts, and neuroendocrine cells. PDEs are cultured submerged or in partial contact with media through a metal grid or collagen/gelatin sponge (Fig. 1). While PDEs retain the structural complexity and heterogeneity of human prostate, they do not include the tumor vasculature nor propagate over time [8, 9]. On the other hand, improved organoid culture methods enabled the 3D tumor cell culture with cells of the TME such as immune cells, carcinoma associated fibroblasts (CAFs), and osteoblasts resulting in a complex architecture representing the cell-to-cell crosstalk in the original tumors (Fig. 1).

#### Methodologies utilized for generating PCa patient derived organoids (PDO)

In spite of the considerable progress in the understanding of the molecular complexity of PCa, culturing PCa cells in vitro has proven challenging. Initial 3D cultures included the spheroid model generated over agarose and later improved in liquid media grown over an agar base [10]. Employing a 3D Rotary Wall Vessel (RWV), PCa cell growth was found to be higher aboard the space shuttle Columbia under micro-gravity simulated condition, when compared to standard conditions on the ground [11]. The similarly inspired hanging drop method allowed 3D culture, without ECM, to form typical 3D spheroids [12] .

The 3D culture of patient derived prostatic epithelial cells in the artificial basement membrane equivalent (BME) matrigel was initially achieved in the presence of serum, dihydrotestosterone, and stomal cells [13]. These culture conditions produced morphological differentiation resulting in acinus like spheroids. Notably, prostate tissue from younger patients with a higher stem-like cell population resulted in greater success in spheroid culture than those from older (> 70 yrs) patients [13]. Matrigel embedded 3D cultures were grown from stem-like cells of hTERT-immortalized nonmalignant and malignant PCa epithelial cells. The CD133^+^ cells in nonmalignant and malignant cell populations retained stem cell patterns of cell behavior, including the high proliferative potential and differentiation ability [14]. While these methods resulted in morphological and functional differentiation of the prostate, their limitations included that serial passaging of cells was not possible.

Pioneering organoid studies in the PDO field have first established intestinal epithelial organoids from Lgr5- expressing stem cells. By incorporating proliferative factors such as the Lgr4/5 ligand and Wnt signaling agonist R-spondin1, BMP signaling antagonist Noggin, and anoikis preventative factor Rho Kinase inhibitor, long-term organoid culture was achieved [15]. Further advances included the use of Rho kinase inhibitor (Y-27632), in combination with fibroblast feeder cells to induce conditional reprogramming of cells (CRCs) [16]. The CRC method enabled rapid and long-term expansion of tumor cells from small biopsy specimens and has been used with variable efficiency in both early- and late-stage PCa. While in culture, CRCs maintain a proliferative phase with the expression of basal, luminal, and stem cell markers. The CRC method also allows for tumor grafting and differentiation in vivo [16].

The various starting material, scaffolds, and culture conditions used in PCa 3D cultures are summarized (Table 1). Several studies utilized matrigel to generate PCa PDOs (Table 1). While matrigel has been a reliable BME, being rich in type-I collagen and laminin, which support the growth and differentiation of nonmalignant and malignant prostate epithelial cells, limitations of matrigel include being animal-derived, inconsistent between patches, and having several undefined factors that could influence organoids growth. A polydimethylsiloxane (PDMS)-based microwell system was also used to culture prostate cells as microaggregates of a controlled size [7]. A novel 3D hyaluronan-base hydrogel system was established to overcome the poor viability of bone metastatic PDX tumor cells. Encapsulated PDX cells can be cultured ex vivo allowing for gene manipulation, drug screening, and investigating PCa bone tropic metastasis. In these and prior reports, the organoids show increased resistance to chemotherapeutic agents. This is attributed to the differential chemical gradients leading to variable drug penetration, as observed in other 3D culture of solid tumors [10]. When compared to their 2D counterparts, organoids also exhibit a lower proliferation rate, which affect their response to chemotherapeutics that target actively dividing cells [10, 32–34]. Encapsulation methods enabled the generation of size-controlled aggregates, in addition to genomic and proteomic analyses used for studying growth kinetics, response to drug treatments, and coculture with cells of TME [35]. Synthetic hydrogels which model the tumor specific ECM niche by crosslinking the ECM ligand polymers to 3D scaffold. Co-culture of these hydrogels with castration-resistant PCa (CRPC) and neuroendocrine prostate cancer (NEPC) PDOs showed a branching morphology, which is consistent with loss of luminal cells in NEPC [35]. Moreover, well-characterized synthetic ECM could be more reliable in predicting drug sensitivities than matrigel [36]. While most PDOs were generated from patient tissue or PDX, few reports followed the initial successful establishment of organoids from circulating tumor cells (CTCs) of metastatic CRCP (mCRPC) patients. To obtain a high yield of CTCs, liquid biopsy apheresis were used [37].

**Table 1:** Culture methods used to generate 3D preclinical models of prostate cancer

Starting material	Media	Serum	Growth factors	Scaffold Basement Membrane Equivalent (BME)	Culture method	Features	Ref no.
**Scaffold-free 3D cultures**							
PCa cell lines (LNCaP, PC3 and DU145)	RPMI 1640 (LNCaP) and DMEM (PC3 and DU145)	10% FBS	None	None	Liquid overlay method	Single aggregates are formed in the well using 1% Seaplaque-agarose with gentle agitation	[10]
PCa cell line (LNCaP) and human osteoblast cell line (MG63) in co-culture	T-medium	10% FBS	None	None	Rotary wall vessel (RWV) culture	Microgravity conditions with perpetual fluid rotation	[11]
PCa cell line (LNCaP)	DMEM	10% FBS	None	None	Hanging drop culture	Non-adherent conditions are created using hanging drop plates sealed with agarose	[12, 17]
PDX from primary PCa	ADMEM/F12 with HEPES, GlutaMAX and Primocin	5% FBS	Y-27632, Nicotinamide, Noggin, Rspondin, N-acetyl-cysteine, DHT, Wnt3a, HGF, EGF, FGF10, FGF2, PGE2, SB202190 (P38 MAP Kinase inhibitor), and A83–01 (TGF-b pathway inhibitor)	None	None	Organoids grown in suspension scaffold-free conditions, with no requirement for ECM support	[18]
**Matrigel-based 3D cultures**							
Non-malignant human prostate tissue	Keratinocyte serum-free medium (KSFM)	None	EGF and BPE	50% Matrigel	Matrigel embedding	Patient stromal cells were cocultured with prostate cells in transwell inserts	[13]
Malignant and nonmalignant human prostate tissue and immortalized cell lines	KSFM	None	EGF, BPE, and R1881 (synthetic androgen agonist)	2% matrigel	Matrigel suspension	Matrigel enabled differentaition of non-malignant prostate epithelial cells and cocultured with irradiated fibroblasts, which were reprogrammed to stem-like cells	[14, 16]
Human prostates from radical prostatectomy and murine prostates	ADMEM F12 with B27, HEPES, Glutamax, and Penicillin/Streptomycin	None	EGF, R-spondin 1, Noggin, A83–01, DHT, FGF10, FGF2, Prostaglandin E2, SB202190, Nicotinamide	100% Matrigel	Matrigel embedding	Optimized conditions for continuous propagation of normal human basal and luminal prostate epithelial cells	[19–27]
Human prostate from radical prostatectomy, PCa cell line (VCaP) and murine prostate	Hepatocyte medium with Glutamax and Penicillin/Streptomycin	5% charcoalstripped FBS	EGF, Y-27632, DHT	5% Matrigel (60% matrigel for drug treatment)	Matrigel suspension Matrigel embedding for drug treatments)	Bipotential Luminal progenitor derived organoids were cultured for upto 13 passages	[28, 29]
PCa cell lines	KSFM for RWPE1 and RPMI-1640 for PCa cell lines	2% FBS	None	50% Matrigel (bottom layer) 25% matrigel (top layer)	Matrigel Sandwich	A comprehensive panel of miniaturized prostate cell culture derived from cell lines was developed	[30]
Patient cells from radical prostatecomy	KSFM	5% charcoalstripped FBS	DHT	50% Matrigel (bottom layer) 33% matrigel (top layer)	Matrigel sandwich	Co-culture with human primary prostate stromal cells improved epithelial organoid viability	[31]
**Synthetic scaffold-based 3D cultures**							
Cell lines (RWPE-1, RWPE-2, and LNCaP)	KSFM	None	EGF and BPE	Polydimethylsiloxa ne (PDMS)	PDMS Microwell platform	Microwell arrays enabled formation of prostate micro aggregates of defined size suitable for HTS	[32, 33]
MDA PCa 118b PDX	ADMEM/F-12 and alpha- MEM medium	2% FBS	None	Thiolated SH: acrylate-PEGGRGDS: acrylate- PEG-PQ-PEGacrylate (4:1:1)	Synthetic organoid culture	PDX tumor cells and osteoblasts were encapsulated together in a 3D hyaluronan (HA) hydrogel. Tumor cell–hydrogel mixture encapsulated in custom-made PDMS molds.	[34]
PCa patient biopsies	ADMEM	None	B27, N-Acetylcysteine, EGF, FGF-10, FGF-basic, A-83-01, SB202190, Nicotinamide DHT, PGE2, Noggin conditioned media and R-spondin conditioned media	PEG-4MAL macromer and cell cross linker at 1:1 ratio	Synthetic organoid culture	Organoids were cultured in synthetic hydrogels that recapitulate PCa ECM	[35]

#### PDO use to identify PCa cell of origin

The adult prostatic epithelium consists of three main cell types: luminal secretory cells, basal proliferative cells, and rare neuroendocrine cells. The ability to form multicellular organoids is a feature of organoid forming cells with multilineage stem-like potential. Basal cells display stem-like higher proliferative, self-renewal, and resistance to androgen depletion properties when compared to luminal cells [19]. Recent single cell RNA sequencing studies suggested that following androgen deprivation, persisting luminal cells could drive prostate regeneration [20]. The initial progress in establishing PCa PDO lines was achieved in 2014 by demonstrating the long-term culture of seven organoid lines derived from biopsies and CTCs and use of PDOs to elucidate PCa initiation [21]. While organoids from biopsies were maintained for 1–2 months, cultures were eventually overtaken by tumor-associated spindle cells or normal epithelial cells from the biopsy material [21]. Utilizing R-spondin1-based organoid culture, organoids demonstrated that both basal and luminal populations contain bipotent progenitors capable of driving basal and luminal differentiation [28]. Notably, while similarly amenable to serial passaging, prostaspheres solely derived from prostate stem cells of basal phenotype fail to exhibit luminal differentiation in the presence of androgens [14]. Other studies demonstrated that prostate organoids can be generated from luminal stem/progenitor cells known as CARNs (castration-resistant Nkx3.1-expressing cells), with functional AR signaling [22]. Transduction of luminal-derived organoids with PCa oncogenic drivers such as Myc/AKT1 produced low-grade prostate adenocarcinomas, whereas the same genetic manipulation in basal cells gave rise to more aggressive tumors in mice with AR loss and PSA expression [23]. In single cell RNA sequencing using mouse and human prostate studies, following castration, the proliferative and self-renewal potential of luminal cells increased [20]. A single basal cell subset (CK5 CK14, p63), and three distinct luminal cell subsets expressing CD24a, CK8 and CK18, were identified. Among the luminal subsets, a predominant L1 subset expressed AR target genes PBSN, NKx3.1, whereas the L2 and L3 subsets expressed Sca1/Ly6a, Tacstd2/Trop2, and PSCA, known to influence stem cell-like properties [20]. Together, these studies improved our understanding of the identity and features of organoid forming cells and supported the use of PDOs for PCa studies. Clonal propagation of single cell-derived organoids combined with single cell sequencing may reveal the cellular pathways contributing to advanced PCa.

#### PDOs capture the genomic and clinical heterogeneity in PCa

Compared to their 2D counterparts, 3D cultures from PCa lines show altered expression of signaling molecules, phospho-proteins, and transcription factors that facilitate 3D growth. Additionally, distinguishing gene expression signatures were identified among morphologically variant organoids, highlighting the advantage of PDOs to model intra- and inter-tumor heterogeneity of PCa [30]. PDOs have also contributed to defining the genomic landscape of advanced PCa. Molecular characterization of the organoid lines revealed TMPRSS2-ERG fusion, PTEN loss, among PCa specific abnormalities, and PDOs demonstrated genomic stability for 6 months when compared to the original genome [21]. Additionally, PDOs offer the ability to introduce secondary genomic alterations using CRISPR/Cas9 or other gene manipulation techniques. Lineage plasticity is a hallmark of aggressive PCa wherein following AR directed therapy, tumor cells evolve from AR dependence and develop NEPC [4, 38]. Genomic characterization of PDOs could define clonal evolution across the sequential PCa stages and/or phenotypes, especially when coupled with longitudinal assessment of molecular vulnerabilities with sequential liquid biopsies. Indeed, advances in liquid biopsy tools allowed the identification of clonal evolution in lethal PCa with complex dynamics and heterogeneity [37].

#### CSC derived PCa PDOs have unique advantages

**Fig. 2:**
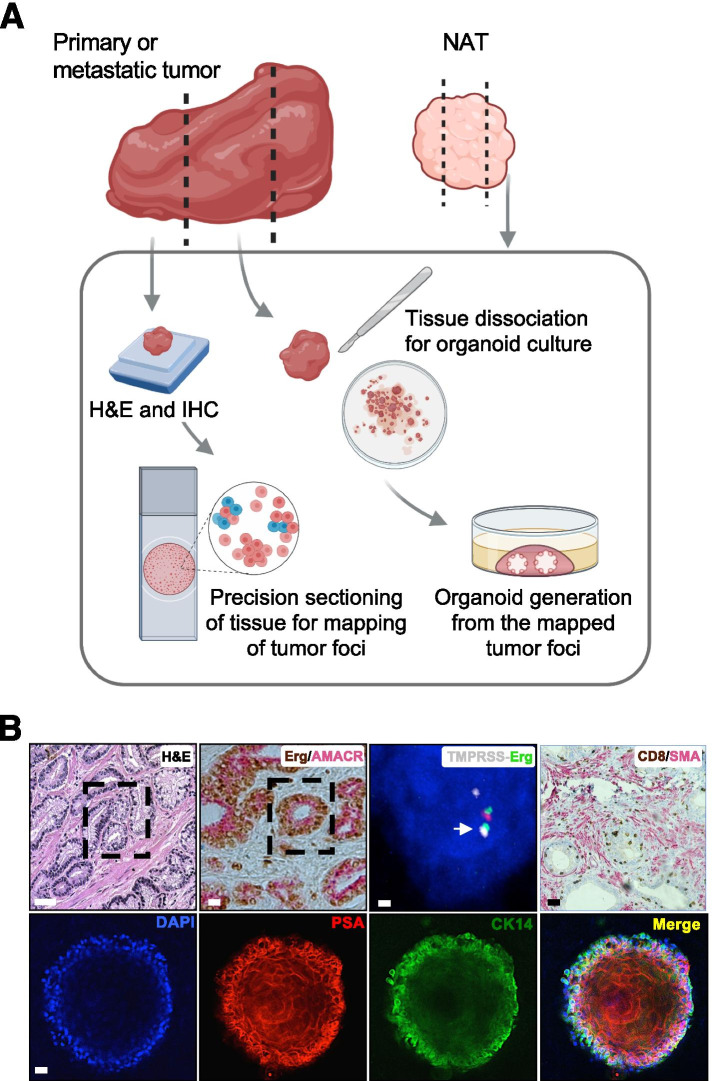
Organoids from primary PCa retain features of originating tumor. **A** Diagram displaying the mapping of tumor foci of patient derived tumors and NAT tissue for successful organoid generation. **B** Representative data from primary PCa PDOs demonstrating retention of the PCa specific genetic alterations such as TMPRSS-Erg fusion. The upper panel demonstrates H&E and IHC staining for Erg (brown) from TMPRSS-Erg fusion co-staining with PCa specific AMACR (red). Fluorescent In Situ Hybridization (FISH) for detecting TMPRSS-Erg fusion. IHC staining could also be used to detect tumor infiltrating lymphocytes (CD8^+^, brown) and tumor microenviromental mapping (SMA^+^, red). The lower panel demonstrates IF staining of single cell derived organoids. The lower panel demonstrates representative images of PDOs derived from single cells and examined on Day 21 for differentiation into lineage specific prostatic cells. PDOs are predominantly showing luminal PSA positive cells (red) and a surrounding layer of basal cytokeratin (CK14, green) localized to periphery. Scale bars are 100 μm in H&E and IF/IHC images and 10 μm in the FISH image

It is noteworthy that most studies reporting successful culture of PCa PDOs had them derived from metastatic and advanced PCa specimens. While several studies reported establishing PCa PDOs from luminal and basal cells, the successful generation of long-term propagating organoids by enriching for CSC has been limited. Tumor initiating cells (TIC) and/or CSC possess tumor initiation and/or self-renewal capacity, drive resistance to therapy, and are considered responsible for tumor recurrence [19]. Organoids derived from single CSC/TIC can be employed to assess tumor progression including the evaluation of metastatic potential and tumor recurrence. We and others have shown that α2β1^hi^/CD44^hi^/CD133^+^ mark distinct TIC, which initiate serially propagating spheres and grafts from primary PCa [19, 39]. When utilizing these cells as organoid forming cells, this approach enables the expansion of CSC and evaluation of single CSC derived organoids. In our laboratory, we have built up on these prior advances and generated primary tumor derived organoids from single multipotent stem-like cells from patients with both localized and advanced PCa (Fig. 2). These PDOs retain the PCa complexity and provide a reliable model to assess drug sensitivity [40], making them an invaluable tool to identify clonal evolution and develop targeted and personalized therapies for PCa. Owing to the association of CSC with drug resistance and tumor relapse, we found that CSC-derived organoids are enriched in B cell-specific Moloney murine leukemia virus integration site-1 (BMI1), a component of Polycomb repressor complex (PRC1). BMI1 regulates stem cell self-renewal through chromatin remodeling and histone modification and pharmacological inhibition of BMI1 inhibited the stem cell-like properties of TIC [41]. Targeting of the stem cell niche to eradicate CSC benefits from the identification of prognostic molecular indicators in organoids such as BMI1, Notch, Oct 3 / 4, Wnt, and Hedgehog pathway modulation.

#### PDOs and the PCa microenvironment

PCa progression is strongly influenced by the surrounding TME, which drives metastasis and regulates response to drugs [42]. Several studies reported the co-culture of non-immune activated stromal cell phenotypes like CAF or endothelial cells with PCa cells. Metastasizing PCa cells are predominantly bone- tropic and 3D co-culture of human PCa with bone cells enables a better understanding of PCa bone tropism and metastasis. The PCa-osteoblast axis was recapitulated in a defined 3D hydrogel system [11, 17]. Additionally, PCa is known to have a predominant infiltrate of mesenchymal stem cells (MSCs), which contribute to disease progression through the generation of CAFs with tumor promoting properties. PCa cells combined with CAFs modeled TME interactions and therapeutic targeting [30]. Moreover, primed CAFs strongly influenced drug response of tumor cells but were eventually outgrown by tumor cells [12]. On the other hand, co-culture with CAFs induced an upregulation of cholesterol metabolism and steroid biosynthesis in PCa cells [31]. More recently, Prostate PDOs were co-cultured with primary prostate stromal cells consisting of fibroblasts and smooth muscle cells. This stromal co-culture induced PCa organoid branching, recapitulated inter-patient heterogeneity, and enhanced organoid viability [43].

**Fig. 3:**
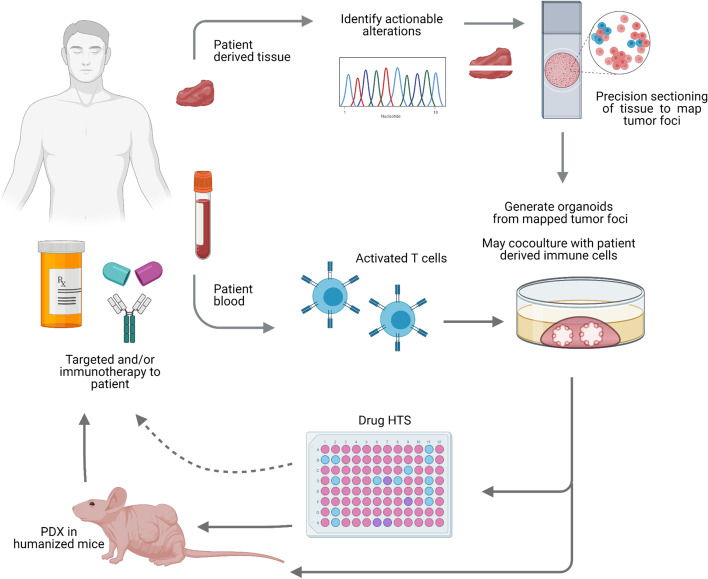
Proposed workflow for the utilization of PCa PDOs for testing treatment efficiency in precision medicine. Patient tumor biopsy or radical prostatectomy tissues are collected and sliced with precision to allow for DNA/RNA sequencing and live cell collection for organoid development. Tumor foci are mapped by H&E from core biopsies prior to radical prostatectomy. Using mirror sections, the region corresponding to mapped tumor foci are used to collect live tissues for generating PDOs. When available, immune cells from the patient blood can be cocultured with tumor cells and/or utilized to generate human immune system in humanized mice. Drug HTS for targeted therapy against actionable mutations can be tested. Simultaneously, PDOs can be engrafted in mice, with or without the human immune system, to validate drug sensitivities and observe the effect of tumor microenvironment. Results can then be translated to the clinic to facilitate precision medicine-based therapies. In the event of tumor recurrence or drug resistance in the patient, the preserved PDOs could be regenerated, validated with liquid biopsy for maintaining parity with the recurrent tumor, and similar or additional drug sensitivity assays can be reinstated to guide the next lines of therapy

Despite observing chronic inflammation in the aging prostate gland and its association with increased risk of aggressive disease, PCa is considered to be of low immunogenicity. Immune checkpoint inhibitors as single agents or in combination have had limited responses in PCa. This could be attributed to the low tumor mutational burden and more so to the PCa unique TME, which is largely comprised of stromal and immune cells with tumor promoting, immunosuppressive profiles [44]. These cell types include regulatory T cells (T-regs), Tumor associated macrophages (TAMs), and myeloid-derived suppressor cells (MDSCs) [44]. An increased understanding of the molecular pathophysiology of PCa and its TME can help to harness more robust anti-tumor responses. A major step in this direction came from a recent study showing that PCa cells prime the immune and non-immune components of the TME infiltrate to facilitate metastasis. In this work, single cell analysis revealed that PCa cells prime infiltrating T cells to express KLK3, the gene encoding PSA. The primed T-cells and TAMs migrate to lymph nodes and bone respectively, creating a pre metastatic niche [45]. Using the air liquid interface method, PDOs from GI cancers were grown as organotypic cultures These PDOs include epithelial, stromal, and immune cells that were maintained for a few weeks, and benefit a detailed investigation of PCa with the TME [46]. In an autologous co-culture model, peripheral blood lymphocytes co-cultured with tumor organoids were enriched for tumor-reactive T cells in colorectal and lung cancers [24]. We envision that utilizing similar approaches could be fruitful in PCa. Currently, we are investigating key growth factors and improved culture conditions to enhance the survival and serial passaging of PCa PDOs and exploring the inclusion of patient derived immune and stromal cells to predict response to targeted therapy and immunotherapy. We are also developing a defined workflow (Fig. 3) using an automated fluidic system to enable high throughput testing of potential therapies for PDOs to be translated to a diagnostic test in a clinically meaningful time frame.

#### PDOs utilization for drug sensitivity assays for precision medicine

A significant advance in employing prostate PDO representative of PCa clinical subtypes for drug testing was reported [18]. A biobank of 20 organoids derived from the LuCaP mCRPC PDX cohort (a PCa PDX cohort designated the LuCaP series), including adenocarcinoma and neuroendocrine lineages, was established. Organoids were grown in 3D culture as well as engrafted as patient-derived organoid xenografts (organoid-PDXs) and subsequently re-passaged in vitro as PDOX-organoids. Notably, Organoids with BRCA2 deficiency displayed sensitivity to the PARP inhibitor olaparib [18]. Similarly, a PDX-derived organoid model from a treatment naïve metastatic PCa was employed for drug screening [47]. These results are encouraging since the DNA damage repair (DDR) genes BRCA1/2, ATM, CDK12, RAD51C and FANKA, are frequently altered in primary PCa and mCRPC [2]. DDR alterations influence the responsiveness of PCa to PARP inhibitors, and BRCA2 reversion mutations are prevalent in patients treated with PARP inhibitors and drive therapeutic resistance [25]. CHD1-deficient PCa might represent a unique molecular subtype with SPOP mutations but lack the TMPRSS-ERG fusions and PTEN deletions. Human PCa derived organoids with CHD1 deletion are hypersensitive to DNA damage and amenable to synthetic lethal response to DNA damaging therapy such as PARP inhibitors [29].

A key implementation of the PDO drug sensitivity assays is to enable targeted therapy in precision medicine, especially for the aggressive PCa subtypes [3]. PCa organoids were genetically manipulated to model a NEPC patient’s genomic alteration in ALK and thereby predicted the response to ALK inhibitors [26]. PDOs were generated from mCRPC patient biopsies with a high success rate (61%) and tested for responses to targeted therapy like BET domain inhibitors [48]. A recent study reported the efficacy of combined RNA polymerase I inhibitor CX-5461 and pan-PIM kinase inhibitor CX-6258 treatment in PDO derived from PDXs of advanced PCa [27]. Apart from various therapeutic targets tested, novel methods to increase the capacity of drug HTS in 3D culture have also been described. For e.g., microwell-meshes can hold up to 150 micro-tumors per well in a 48-well plate for drug HTS [33]. A detailed protocol for establishing PDOs from epithelial tissues including the prostate has been reported [49]. A high rate of success for PDO generation is a pre-requisite for their inclusion in precision medicine clinical trials [3]. Most protocols describe the generation of PDOs from whole tissues. Alternatively, patient tissues can be divided into mapped sections for molecular analysis, histochemistry, and organoid culture [40]. Immunohistochemistry of patient tissue can guide mapping of tumor foci and mapped tumor rich foci can be selected for clonal organoid culture (Fig. 3). The limitations to the implementation of this strategy include the size and availability of tissue biopsies.

#### Inclusion of PDOs in clinical trials

Several ongoing global clinical trials are employing organoids in correlative studies, including NCT03952793 in France, NCT04723316 in UK, NCT02695459 in Netherlands, NCT04927611 in China, NCT03896958 (Precision Insights On N-of-1 Ex vivo Effectiveness Research) in Georgia in the US, and our center trial NCT02458716 at Rutgers Cancer Institute of New Jersey in the US. In a positive advance towards including PDO-based drug sensitivity assays for precision medicine, a Phase II trial of Aurora Kinase Inhibitor Alisertib (MLN8237) included examining organoids from Patients with mCRPC and NEPC. Response to Alisertib in PDOs was in line with patient clinical response data. PDOs also enabled testing for on-target activity of the drug through assaying Aurora–N-Myc complex disruption [50]. While it is encouraging that PDOs are being implemented in clinical trials, the number of trials is still limited. This is mostly attributed to the lack of access to tumor tissues. With the recent advances in prostate PDO culture methods utilizing single cells, one can hope for an improved efficiency of PDO generation that translates to improving therapeutic efficacy predictions and clinical decision making.

## Conclusion and perspective

The past decade has seen significant advances in PCa research. With the increased understanding of the origin and the molecular landscape of PCa, there has been an encouraging trend of precision medicine-based approach to treat advanced PCa. PDOs enabled a better comprehension of the complexity of PCa initiation and progression. With the advances in drug HTS employing PDOs, these advances have catapulted PDOs as a mainstay pre-clinical model. There still remains some barriers for their widespread use to predict treatment responses. The key limitation is the lack of stromal, immune, and endothelial cell components in established PDO cultures. To implement PDOs benefiting precision medicine in PCa, an interdisciplinary approach is required. This approach involves the use of 3D tissue engineering, large scale genomic and morphological profiling, engraftment of PDOs in humanized mice, and longitudinal assessment with liquid biopsies to evaluate targeted therapy effects in the host microenvironment [40]. We envision that the development of novel approaches allowing the co-investigation of tumor cells and TME in PDOs will further advance the applications of PDOs in clinical medicine.

## Data Availability

Not applicable.

## Abbreviations

2D: Two-dimensional
3D: Three-dimensional
ADT: Androgen deprivation therapy
CAF: Carcinoma associated fibroblast
CSC: Cancer stem-like cell
CRPC: Castration resistant prostate cancer
CTC: Circulating tumor cell
FISH: Fluorescent in situ hybridization
HTS: High throughput screening
IF: Immunofluorescence
IHC: Immunohistochemistry
mCRPC: Metastatic castration resistant prostate cancer
PDO: Patient derived organoids
PCa: Prostate Cancer
PDE: Patient derived explant
PDX: Patient derived xenograft
TIC: Tumor initiating cell
TME: Tumor microenvironment
